# Influence of point mutations on PR65 conformational adaptability: Insights from molecular simulations and nanoaperture optical tweezers

**DOI:** 10.1126/sciadv.adn2208

**Published:** 2024-05-31

**Authors:** Anupam Banerjee, Samuel Mathew, Mohsin M. Naqvi, Sema Z. Yilmaz, Maria Zacharopoulou, Pemra Doruker, Janet R. Kumita, Shang-Hua Yang, Mert Gur, Laura S. Itzhaki, Reuven Gordon, Ivet Bahar

**Affiliations:** ^1^ Laufer Center for Physical and Quantitative Biology, Stony Brook University, Stony Brook, NY 11794, USA.; ^2^ Department of Electrical and Computer Engineering, University of Victoria, Victoria V8P 5C2, Canada.; ^3^ Department of Pharmacology, University of Cambridge, Tennis Court Road, Cambridge CB2 1PD, UK.; ^4^ Department of Mechanical Engineering, Istanbul Technical University, 34437 Istanbul, Turkey.; ^5^ Department of Computational and Systems Biology, School of Medicine, University of Pittsburgh, Pittsburgh, PA 15213, USA.; ^6^ Department of Electrical Engineering, National Tsing Hua University, Hsinchu 30013, Taiwan.; ^7^ Department of Biochemistry and Cell Biology, School of Medicine, Stony Brook University, Stony Brook, NY 11794, USA.

## Abstract

PR65 is the HEAT repeat scaffold subunit of the heterotrimeric protein phosphatase 2A (PP2A) and an archetypal tandem repeat protein. Its conformational mechanics plays a crucial role in PP2A function by opening/closing substrate binding/catalysis interface. Using in silico saturation mutagenesis, we identified PR65 “hinge” residues whose substitutions could alter its conformational adaptability and thereby PP2A function, and selected six mutations that were verified to be expressed and soluble. Molecular simulations and nanoaperture optical tweezers revealed consistent results on the specific effects of the mutations on the structure and dynamics of PR65. Two mutants observed in simulations to stabilize extended/open conformations exhibited higher corner frequencies and lower translational scattering in experiments, indicating a shift toward extended conformations, whereas another displayed the opposite features, confirmed by both simulations and experiments. The study highlights the power of single-molecule nanoaperture-based tweezers integrated with in silico approaches for exploring the effect of mutations on protein structure and dynamics.

## Teaser

Optical tweezers and molecular simulations reveal differential conformational adaptability due to hinge site mutations in PR65.

## INTRODUCTION

Maintaining cellular signaling and homeostasis is crucial for the proper functioning of living organisms, and dysregulation of these processes can result in the development of many diseases. A complex interplay between kinases and phosphatases contributes to signaling events and cellular homeostasis (*1*). Abnormal activation of kinases and inactivation of phosphatases can lead to pathological hyperphosphorylation, a key factor in the development of numerous diseases, including cancer and neurodegenerative disorders (*2*, *3*). Much attention has been given to kinase inhibitors for the treatment of these diseases. Phosphatases, on the other hand, have been much less studied as drug targets (*4*–*9*) mainly due to the lack of druggable pockets near their active sites (*10*, *11*).

One major class of phosphatases playing a central role in maintaining cellular homeostasis is the family of serine/threonine protein phosphatases 2A (PP2A) (*12*–*14*). PP2A is frequently dysregulated in human diseases, making it an attractive target for therapeutic interventions (*15*). It is a heterotrimer, composed of a scaffold (A) subunit, known as PR65, a catalytic (C) subunit, and one of many regulatory (B) subunits. The A and C subunits form the core of PP2A.The specificity of PP2A is controlled by the choice of the regulatory subunit that binds the AC core, with over 40 different B subunits each determining the specific substrate bound to PP2A (*3*). The diverse array of B subunits allows PP2A to exert control over a majority of cellular signaling pathways.

PR65 serves as a structural scaffold that provides a platform for the assembly of the heterotrimer (*16*). Among the three PP2A subunits, PR65 experiences the highest frequency of mutations, which have been implicated in altering PP2A activity (*17*–*19*). Understanding the impact of PR65 point mutations on PP2A structure and function is essential to unraveling the mechanisms underlying various diseases and developing targeted therapeutic strategies (*20*). PR65 is a tandem repeat (TR) protein consisting of 15 HEAT repeats, each comprising ~40-residue antiparallel helices. These repeats stack in a one-dimensional fashion, forming an elongated, horseshoe-like superhelical structure composed of outer and inner helices layers. Many TR proteins act as hubs in multiprotein complexes, whereby their conformational fluctuations facilitate the function of the assembly (*21*–*23*). In the case of PR65, its mechanics play a crucial role in regulating PP2A function; the collective motions of PP2A mediated by PR65 open and close the enzyme’s substrate binding/catalysis interface. Maintaining the flexibility of PR65 to sample alternative conformers is crucial, with the closed state being active and facilitating the formation of the PP2A complex with catalytic and regulatory subunits (*24*, *25*). Although the effects of PR65 mutations on folding and binding have been studied (*17*–*20*), a systematic investigation of the modulation of PR65’s conformational state, flexibility, and adaptability by point mutations is lacking. Here, we aim to fill this gap. We focus on a unique aspect of PR65 structural mechanics—the so-called “hinge” sites that coordinate the global dynamics of the entire complex. Hinge regions usually play a key role in mediating the conformational mechanics of the proteins and enabling conformational changes that underlie functional transitions (*26*).

By specifically targeting mutations at the hinge sites, we aim to investigate how subtle alterations in these regions influence the conformational space accessible to PR65. Our approach is to examine the changes in the conformations and dynamics of PR65 induced by introducing point mutations at those sites. We hypothesize that certain mutations may restrict the conformational adaptability of PR65, potentially impacting the formation of the PP2A complex and its activity. To assess the effect of point mutations, we integrated molecular dynamics (MD) simulations and elastic network model-based analysis (*27*) and experimentally tested the findings using nanoaperture optical tweezer measurements (*28*).

An outstanding challenge in biology is how to visualize protein structure at the single-molecule level and on functionally relevant timescales without the use of labels, immobilization, or tethering, which can result in artifacts that perturb the system we are trying to observe (*29*). Here, we present nanoaperture optical tweezers. Optical tweezers have emerged as a powerful tool for probing the biophysics of proteins at the molecular level. The present study demonstrates the utility of the enhanced field confinement and sensitivity provided by nanoaperture-based tweezers for studying the structure and dynamics of single proteins. Unlike conventional optical tweezer techniques, this approach allows for characterization of individual unmodified proteins in solution for extended durations and without the need for tethers or labels (*30*, *31*).

Here, we focused on hinge sites of PR65 and selected, with the help of site-directed saturation mutagenesis in silico (*32*, *33*), six point mutations: Y168V, L197V, D315E, S323L, E375D, and F502W. Extensive MD simulations revealed that S323L, E375D, and F502W favored the adoption of relatively more extended conformations compared to the wild type (WT), while the conformational flexibility enabling the fluctuations between open and closed conformers was relatively suppressed. The Y168V and D315E mutants, in contrast, favored relatively more compact conformations with mixed effects on conformational dynamics. The F502W mutation helped to regulate the transitions between the compact and extended form in a more favorable way than the WT PR65. The overall stability and dynamics of D315E, on the other hand, was perturbed, interfering with the ability of PR65 to adapt to conformational changes required for function. Concurrently, by trapping the mutants in the optical tweezer setup, we measured their motion and observed that the mutants S323L and F502W shifted toward extended conformations. The more extended conformations were indicated by smaller signal–root mean square deviations (RMSDs) in their amplitudes of motions along with higher corner frequencies measured in nanoaperture optical tweezer experiment. In contrast, the signal-RMSD for E375D was larger compared to WT PR65. Since elongation typically leads to a higher polarizability (*34*) and therefore larger optical tweezer stiffness, these findings independently confirm the conformational variability for all six mutants.

**Fig. 1.**
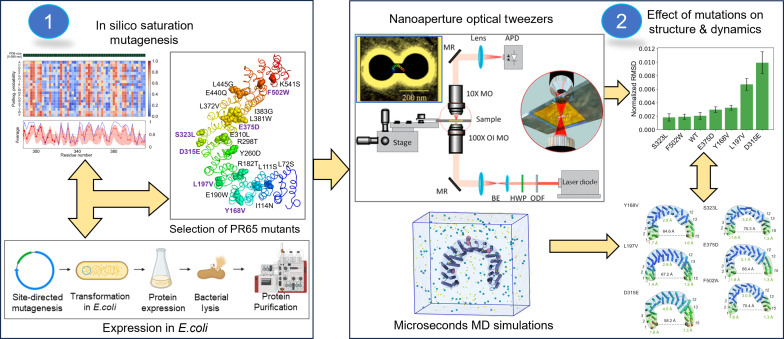
Schematic description of the integrated experimental and computational methodology. (1) Selection of PR65 mutations and experimental validation of their thermodynamic stability: Residues that act as hinge sites across the six most cooperative modes of motion of PR65 were identified using the Gaussian network model (GNM), based on the hypothesis that mutations at those sites would alter PR65 conformational flexibility. We first checked that the mutations would not destabilize the scaffold. Small-scale protein expression tests in *E. coli* conducted for 21 mutants identified six mutants that showed good expression. Further large-scale expression of these six mutants, and thermal unfolding, confirmed that their thermodynamic stability was not compromised. (2) Analysis of the conformational flexibility of the PR65 mutants: The mutants were analyzed by nanoaperture optical tweezer experiments and MD simulations to determine their effect of mutations on structure, dynamics, and conformational adaptability. See further details in Materials and Methods and in Results.

Overall, our study represents an integration of results from in silico saturation mutagenesis, MD simulations, and optical tweezer experiments, as schematically described in Fig. 1 to characterize the conformational mechanics of PR65 and its mutants. The combination of these complementary approaches provides insights into the modulation of PR65 conformational flexibility and adaptability, laying the foundation for further investigations and potential therapeutic interventions. It also offers an integrated computational and experimental protocol for exploring the structural and dynamic consequences of mutations, generalizable to other systems.

## RESULTS

### In silico saturation mutagenesis of PR65

**Fig. 2.**
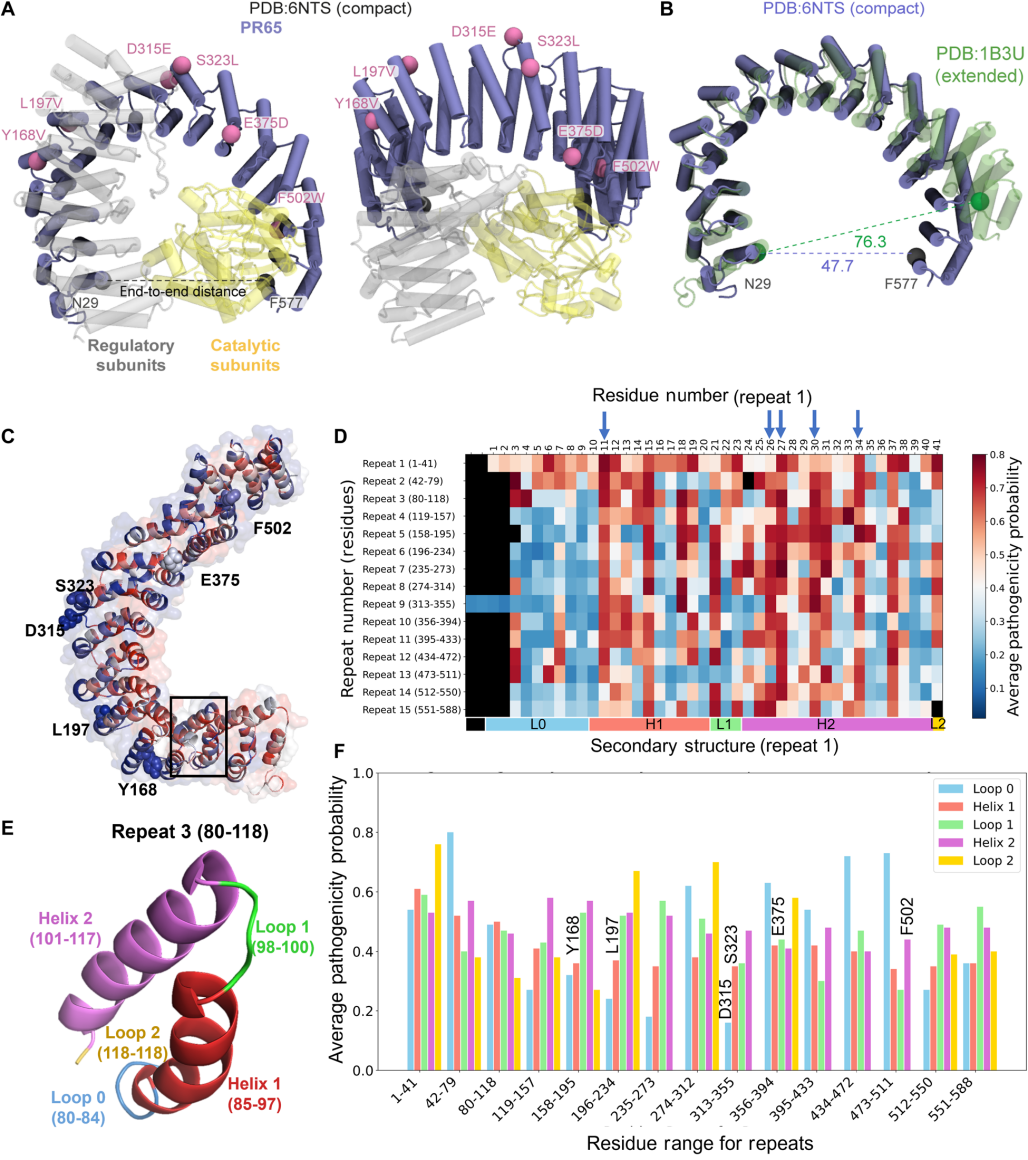
PP2A heterotrimer, conformational adaptability, and potential pathogenicity of its scaffolding subunit PR65. (**A**) Two different views of the PP2A trimer [Protein Data Bank (PDB): 6NTS (*3*)] with PR65 shown in navy/mauve, and the catalytic and regulatory subunits in yellow and gray, respectively. The locations of six residues mutated in the present study are indicated by beads. (**B**) Compact (solid) and extended [green, transparent; PDB: 1B3U (*78*)] conformations of PR65 in the trimer and in isolation, respectively. N29 and F577 are used as references to define the end-to-end distance (*37*). (**C**) PR65 color-coded by average pathogenicity of residues as predicted by Rhapsody. The color ranges from blue (lowest probability of being deleterious; 0.12 here) to red (highest probability; 0.80). Note that mutations at the inner portions of the repeats generally tend to have more deleterious effects compared to the outer regions. (**D**) Heatmap showing the average pathogenicity of residues (abscissa) within each of HEAT repeat (ordinate). Nonexistent residue positions are shown in black. (**E**) Loop0-helix1-loop1-helix2-loop2 motif of HEAT repeats, illustrated here for repeat 3. Helix 1 is outer, and helix 2 is inner. (**F**) Average pathogenicity probability for the helices and loops of each HEAT repeat. Consistent with (A), helix 2 tends to exhibit higher sensitivity to mutations than helix 1, whereas the inter-repeat loops 0 and 2 exhibit higher pathogenicity than the intra-repeat loop 1. The color-coded secondary structure corresponding to the first repeat is shown along the abscissa (bottom) of (B). Note that not all repeats have the same helix and loop lengths.

As illustrated in Fig. 2A, PP2A is a heterotrimer composed of subunits A (PR65), B (regulatory), and C (catalytic). The PR65 subunit has been resolved in both open/extended and closed/compact forms (Fig. 2B). The in silico saturation mutagenesis study of PR65 was performed using a recently introduced structure- and dynamics-based machine learning methodology, implemented in the online accessible tool Rhapsody (fig. S1) (*32*, *35*). The approach allows for assessing the impact, neutral or pathogenic, of any substitution at any residue along the protein based on sequence (conservation and coevolution) (*36*), structure (accessible surface area), and dynamics (equilibrium fluctuations, allosteric couplings, and mechanical behavior) of the protein. In parallel, we estimated the change in folding free energies associated with point mutations using PROTSPOM (fig. S2A) (*33*). PROTSPOM uses residue physicochemical and energetic properties in the folded state, environmental compatibility, and evolutionary information to predict the change in Gibbs free energy (ΔΔ*G*) of folding associated with point mutations.

Figure 2 (C to F) presents the results for the apo structure of PR65. The diagram in Fig. 2C is color-coded by average pathogenicity score for each residue *i*, i.e., the probability of having a deleterious/pathogenic effect upon mutating the *i*th residue, averaged over all 19–amino acid substitutions at that position. The effects of the individual substitutions are described by the elements of the *i*th column in the saturation mutagenesis heatmap (see fig. S1 for a PR65 segment). The scores vary from 0 (neutral, blue) to 1 (strongly pathogenic, red), with a cutoff of 0.60 determining the decision between neutral and deleterious. Using the residue ranges defined earlier (*37*), we evaluated the pathogenicity scores of the residues within each of the 15 HEAT repeats. The results are presented in Fig. 2D, organized by repeat number (ordinate) and corresponding residue positions (abscissa). The heatmap shows that the counterparts of the repeat 1 residues P11 (in helix 1), L26, R27, S30, and L34 (in helix 2) in all repeats consistently exhibit high pathogenicity probabilities. These residues are indicated by blue arrows along the abscissa. Their resistance to tolerate mutations is consistent with their high degree of sequence conservation at those positions, usually occupied by hydrophobic residues (leucine, valine, and isoleucine) or by arginine. See the counterpart of this heatmap corresponding to change in free energy of folding, ΔΔ*G*, in fig. S2B.

Closer examination of HEAT repeat structural elements (loop 0, helix 1, loop 1, helix 2, and loop 2; Fig. 2E) revealed the distinctive behavior of inter- and intra-repeat elements. In Fig. 2F, we present the average pathogenicity for these structural elements. The corresponding residue ranges are listed in table S1 (*37*). Notably, the loops 0 and 2 linking successive repeats generally exhibit relatively high pathogenicity if mutated, suggesting the high sensitivity of PR65 if not inability to tolerate mutations at inter-repeat regions. See the peaks in Fig. 2F between repeats 1 and 2, and those at loop 0 or 2 between repeats 6 and 7, 8 and 9, 10 and 11, 11 and 12, and 12 and 13. The latter two represent kinks of single residues (G434 and G473, respectively), rather than loops, that presumably play a critical role. The counterpart of this analysis for ΔΔ*G* is presented in fig. S3, which also draws attention to the critical role of S119 between repeats 3 and 4.

This analysis therefore identified the inter-repeat loop residues to generally play a critical role in ensuring the overall stability and/or functional flexibility of PR65. Closer analysis also identified specific mutations at the regulatory and catalytic subunit interfaces of PR65 that would induce the strongest destabilization and pathogenicity. Table 1 lists these mutations, and fig. S4 displays their location in the PP2A structure. We note again the propensity of helix 2 residues among these critical sites.

**Table 1.** PR65 mutations distinguished by highly destabilizing and/or pathogenic effects.

Location	Mutation	Repeat no., secondary structure	ΔΔ*G* (kcal/mol)	Pathogenicity score
Interface with the regulatory subunit	M179H	Repeat 4, helix 2	1.52	0.71
	R182T	Repeat 5, helix 2	1.46	0.77
	S255F	Repeat 6, loop 1	1.47	0.86
	W256H	Repeat 6, helix 2	2.08	0.82
Interface with the catalytic subunit	W416F	Repeat 11, helix 2	1.23	0.82
	R497T	Repeat 13, helix 2	1.34	0.81

### In silico evaluation of potential mutations at hinge sites

**Fig. 3.**
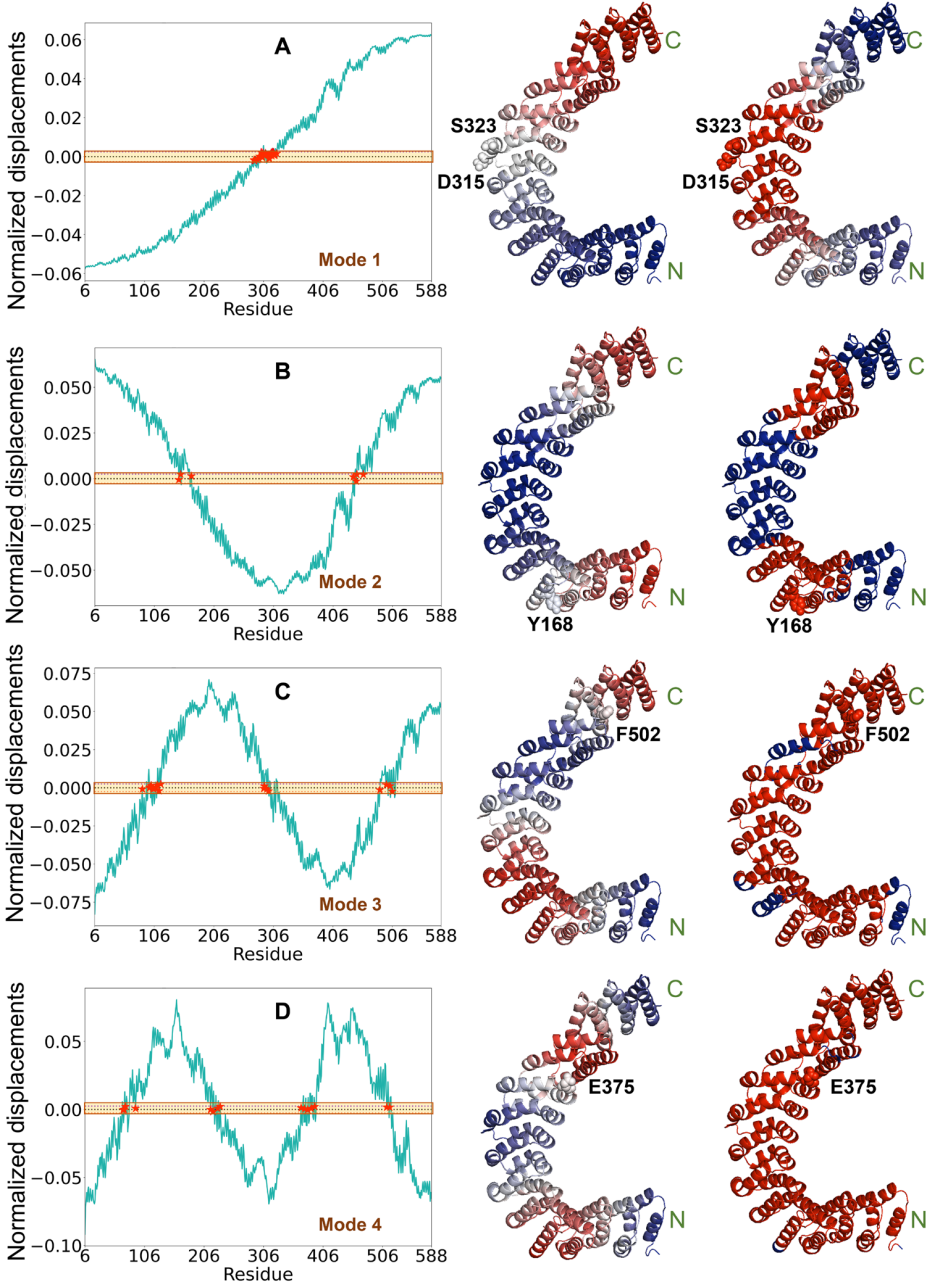
Hinge residues facilitate conformational transformation of PR65. (**A**) Results for mode 1. The left curve shows the normalized displacements of PR65 residues along GNM mode 1. The region within the band 0 ± 0.0025 (shown in yellow shade) indicates the crossover between positive and negative direction motions along the mode axis, and the residues lying in this region (shown by red stars) act as global hinge sites. The middle ribbon diagram is color-coded according to the displacement along this mode, varying from blue (negative) to red (positive). Hinge sites are at the crossover between blue and red regions. The right diagram is color-coded by the size of the displacements from red (minimum displacement) to blue (maximum). Hinge sites are colored red, as they are subject to minimal displacements if any. Selected hinge site residues are labeled in each case. The corresponding mutants (listed in table S2) were found to be expressed and soluble in substantial amounts, enabling further investigation of their impact on PR65. The same results are presented for GNM modes 2 to 4 in (**B**) to (**D**), respectively.

With the objective of exploring point mutations that alter the conformational state and dynamics of PR65, we focused on hinge residues that play a dominant role mediating the conformational mechanics of the protein. The Gaussian network model (GNM) analysis (*38*, *39*) permits us to identify residues that control the softest (most cooperative) modes of motion accessible to the examined protein. Figure 3 (A to D) shows the shapes of the GNM softest modes 1 to 4 (left) predicted for PR65 as a function of residue index. The crossover between negative and positive regions along the mode axis (indicated by red stars) defines the hinge sites corresponding to each mode. The hinges are located at C309-S323 in mode 1, near Y168 and E440 in mode 2, L111, R298, and F502 in mode 3, and L72, D217, E375, and F502 in mode 4. The ribbon diagrams in Fig. 3 are color-coded by the direction (middle) and size (right) of residue displacements along those modes. We have additionally considered a few hinges from modes 5 and 6 (not shown) to select a total of 21 hinge residues (listed in column 2 of table S2).

Next, we proceeded to the evaluation of all possible amino acid substitutions at those 21 residues to select those specific mutations that would not completely abolish the function or stability of the protein. Toward this goal, we analyzed the results from the two sets of computations described above: (i) in silico saturation mutagenesis using the interface Rhapsody (*32*, *35*) and (ii) folding free energy change ΔΔ*G* between the mutant and WT PR65 using PROTSPOM (*33*). In the interest of exploring mutations that would induce distinctive effects, we considered a broad range of pathogenicity scores and ΔΔ*G* values and selected those mutations that yielded consistent results between the two tools. We categorized the mutants into two broad groups based on their Rhapsody scores: (a) pathogenic and (b) nonpathogenic. This resulted in nine nonpathogenic mutations, highlighted in bold in table S2, which we estimated to retain the fold while altering the conformational state and dynamics. We further divided this group into three subgroups based on their ΔΔ*G* values: (b1) not destabilizing (ΔΔ*G* < 0), (b2) mildly destabilizing (0 ≤ ΔΔ*G* < 1.25 kcal/mol), and (b3) highly destabilizing ΔΔ*G* ≥ 1.25 kcal/mol. The solvent accessibility (*40*) and sequence conservation (*41*) of the mutated residues are also provided in table S2. All mutations were subjected to protein expression tests, which led us to select six, as explained next.

### Experimental assessment of the stability of in silico–suggested mutants to select six mutants

We previously used *Escherichia coli* to produce recombinant PR65 WT and mutants for folding studies (*20*). To assess the thermodynamic stabilities of the mutants in table S2, we first examined the 12 mutations in group (a), which were predicted to adversely affect the protein or function (pathogenicity score > 0.60). Most of these mutations were also predicted to be highly destabilizing (ΔΔ*G* ≥ 1.25 kcal/mol). We performed small-scale protein expression tests in *E. coli* on them, and the results showed that 11 of these mutants had no expression or were insoluble, consistent with the computational predictions. Experiments repeated for the nine nonpathogenic mutants, on the other hand, showed that eight (except E392Q) were expressed, six of them in good amounts, and two in small amounts. We focused on these six mutants for further studies. Notably, three of them (D315E, S323L, and E375D) belonged to group b1, one (F502W) to subgroup b2, and two (Y168V and L197V) to subgroup b3.

We next performed large-scale expression of these six mutant proteins and used thermal unfolding to qualitatively assess their thermodynamic stabilities as measured by melting temperature (the temperature at which the protein is 50% unfolded) (table S3). All mutants had melting temperatures within 1°C of the WT value (51.3°C), indicating that the mutations had only very small effects on stability. We therefore moved on to examine the impact of these six hinge site point mutations (Y168V, L197V, D315E, S323L, E375D, and F502W) on the structure, dynamics, and potentially function of PR65 by MD simulation and optical tweezer experiments.

### MD simulations indicate that the mutants S323L, E375D, and F502W preferentially sample extended conformations

**Fig. 4.**
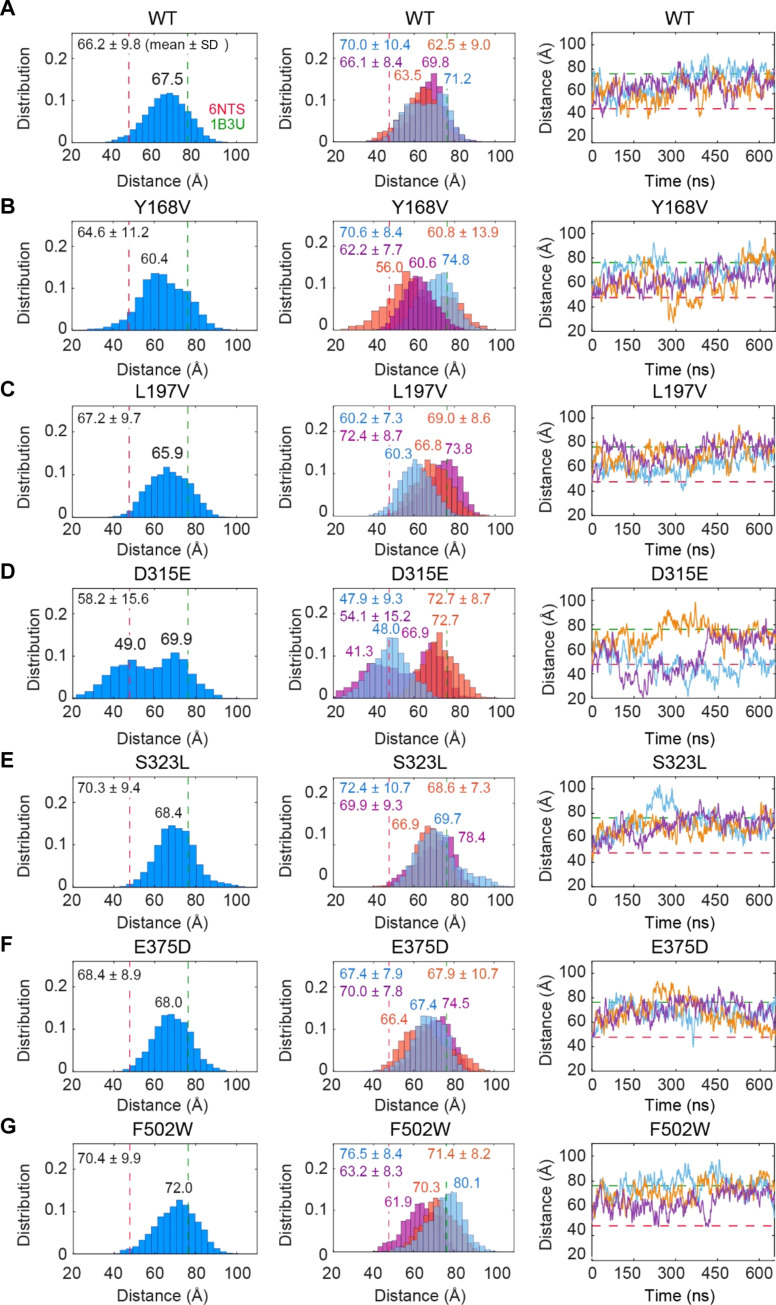
The mutants S323L, E375D, and particularly F502W sample more extended conformations than WT PR65. (**A**) End-to-end distance distributions and time evolution for WT PR65. Left and middle panels show the distribution of end-to-end distances for the combined MD trajectory (blue) and each set of simulations separately (light blue, magenta, and orange), respectively. The mean ± SD is indicated in each histogram. Location of peaks is indicated on the distributions. Dashed lines represent the end-to-end distances observed in the resolved compact (PDB: 6NTS) and extended (PDB: 1B3U) structures of PR65. The right panel shows the time evolution of the end-to-end distance. (**B** to **G**) Same as (A) for the indicated mutants.

Simulations were initiated from the compact form of PR65, taken from the heterotrimeric PP2A (*3*). The distributions of the end-to-end distances, defined as the distance between the C^α^ atoms of N29 and F577 [as in earlier work (*37*)], are presented in  Fig. 4 (A for WT and B to G for the six mutants). In each case, the cumulative histogram deduced from triplicate runs is shown in the left panel, and the individual histograms from each of the three runs are shown in the middle. The mean values and standard deviations (SDs) are written in each case, and their averages over triplicate runs are reported in Table 2 (columns 3 and 4). The panels on the right of Fig. 4 show the time evolution of the end-to-end distance for each run. Consistent with our previous study (*16*), WT PR65 (Fig. 4A) samples a broad range of end-to-end distances, from 36.3 to 95.8 Å, including those resolved for the compact (47.7 Å; see Fig. 2B) and extended structures (76.3 Å), with a mean value of 66.2 Å. All three independent runs consistently exhibited similar distributions.

**Table 2.** Pathogenicity and conformational dynamics of the six selected mutations.

Mutant	Results from theory and computations			Results from nanoplasmonic optical tweezer experiments	
	Pathogenicity score [0–1]*	Average end-to-end distance (Å)	SD in end-to-end distance (Å)	Corner frequency (Hz)^†^	Normalized signal-RMSD^‡^
				(mean ± SD)	
S323L	0.24	70.3	9.4	122.82 ± 5.4	0.0018 ± 0.0009
F502W	0.36	70.4	9.9	45.07 ± 2.0	0.0019 ± 0.0007
WT	0	66.2	9.8	24.66 ± 2.9	0.0021 ± 0.0009
E375D	0.20	68.4	8.9	25.75 ± 5.9	0.0030 ± 0.0007
Y168V	0.18	64.6	11.2	20.97 ± 4.3	0.0033 ± 0.0006
L197V	0.04	67.2	9.7	16.78 ± 1.2	0.0067 ± 0.0015
D315E	0.03	58.2	15.6	15.99 ± 3.1	0.0099 ± 0.0028

*All mutants are nonpathogenic, their scores being below the threshold 0.6.

†Higher corner frequency indicates stiffer trapping or more extended/stretched conformations.

‡Signal-RMSD increases with increasing conformational variability or decreasing corner frequency.

The triplicate runs conducted for each of the mutants S323L, E375D, and F502W also showed overlapping distributions of end-to-end distances despite small shifts (middle histograms in Fig. 4, E to G). However, the main difference from the WT PR65 was the shifts in the end-to-end distances toward more extended states. The corresponding mean end-to-end distances (70.3, 68.4, and 70.4 Å, respectively; cumulative histograms on the left) are larger than that of the WT. Thereby, these mutations favor more extended conformations in comparison to the WT PR65.

In contrast, the mutants Y168V and L197V (Fig. 4, B and C) were observed to sample end-to-end distances comparable to that of the WT PR65, if not more compact forms. D315E was able to sample much more compact conformations (as low as 17.5 Å) than WT and gave the lowest mean end-to-end distances. For Y168V, L197V, and D315E, the main effect of mutations seems to compromise the ability of the structure to uniformly sample the conformational space; instead, the individual runs tend to gravitate/drift toward different forms, as evidenced by the histograms (middle diagrams) that show only a partial overlap. This effect was particularly pronounced in the mutant D315E, where two of the runs sampled rather compact forms with new peaks appearing at end-to-end distance of 41.3 and 48.0 Å, whereas the third run sampled an extended form (mean value of 72.7 Å) with no transition to the compact form (Fig. 4D).

### Nanoaperture optical tweezer–based characterization of PR65 and its variants

Optical tweezers have been used widely to probe the biophysics of proteins at the single-molecule level (*42*–*44*). By using enhanced field confinement and sensitivity, nanoaperture-based plasmonic tweezers have been adopted by several groups to study single proteins, protein complexes, and their interactions without the need for (or potential impact from) tethers or labels (*30*, *31*, *45*–*49*). Furthermore, since the first work of trapping single proteins with double nanoholes (DNHs) (*30*), there have been several works that convincingly showed that nanoaperture-based plasmonic tweezers provide single-molecule measurements (*50*). For example, it was demonstrated that combining the DNH with a nanopore, which has a clear single-molecule signal, the observed signal of the DNH was consistent with single-molecule events (*31*). It has also been demonstrated that in the rare occasion of multiple proteins being trapped, a larger change in the optical transmission is observed (*51*). Finally, molecular weight sizing of proteins was achieved by monitoring the transmission signal, and this was consistent with single protein sizes (*52*). Here, we trap the protein using double nanoholes (*53*) fabricated by a random colloidal lithography technique (*28*), as schematically illustrated in part 2 of Fig. 1. A 980-nm laser with a 1.3 NA (numerical aperture) 100× objective is focused on the aperture, with 22.5 mW of power incident on the aperture in a diffraction limited spot. The transmission through the aperture is monitored on an avalanche photodiode [using a 1.3–optical density (OD) filter to prevent saturation].

**Fig. 5.**
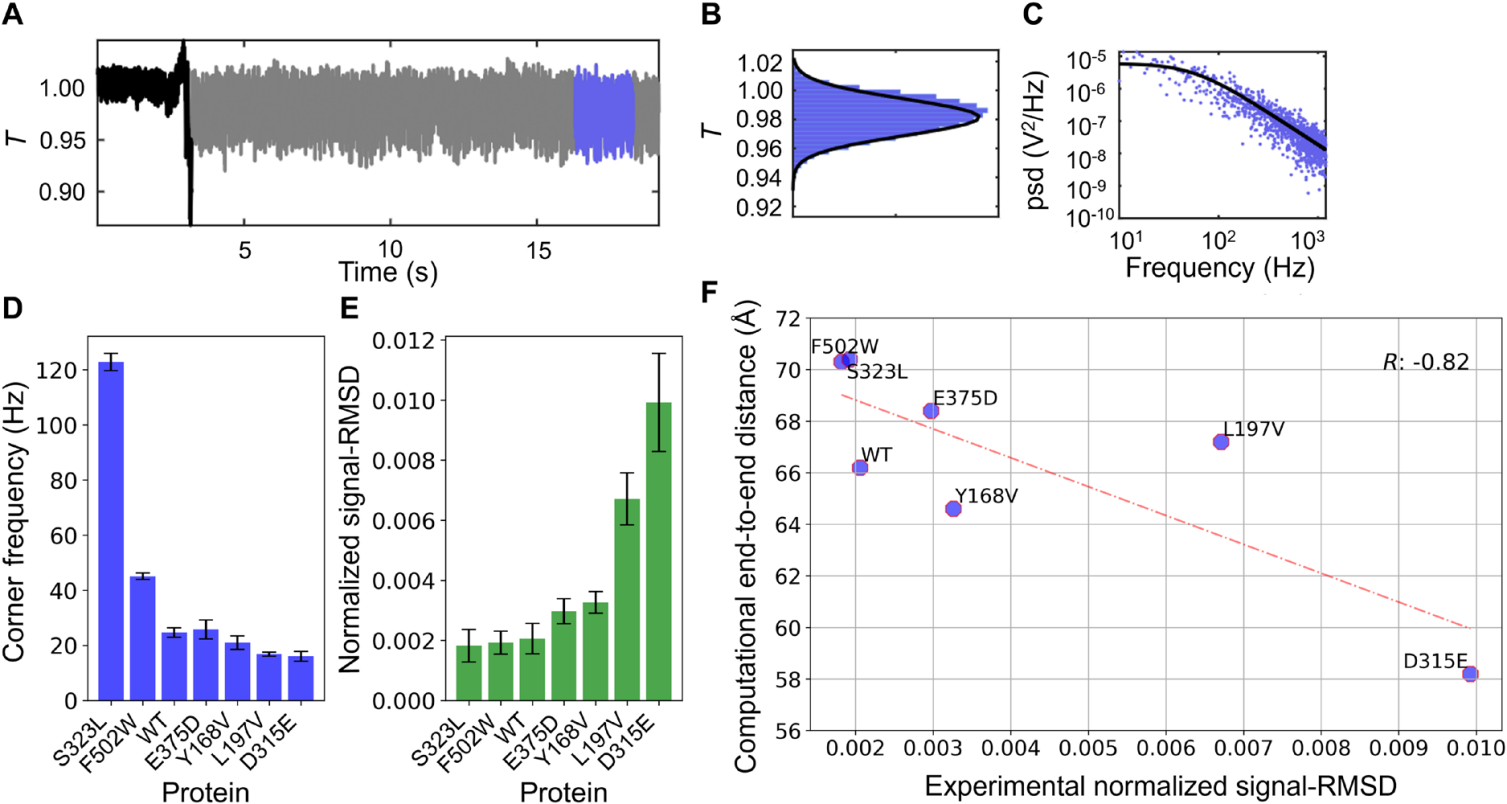
Tetherless aperture-based nanoplasmonic tweezers enable characterization of the structure and dynamics of PR65 and single-site mutants. (**A**) PR65 undergoes a characteristic step as it enters the double nanohole trap, followed by an increase in noise from Brownian translational motion. (**B**) Histogram of this noise (transmission through the aperture, *T*, normalized to the pre-trap level) sampled after the trapping event. (**C**) Power spectral density from the detected transmission of PR65. (**D**) Corner frequency of the Brownian motion of WT PR65 and six mutants in trapping experiments. (**E**) Signal-RMSD measured for the six mutants and WT PR65. Note that the signal-RMSD decreases with increasing stiffness/polarizability/extension of the trapped molecules such that more extended proteins give rise to smaller signal-RMSD. (**F**) Comparison of the average end-to-end distance observed in 1.962-μs MD simulations for each of the seven investigated proteins (WT PR65 and the six mutants) and the signal-RMSD measured in nanoplasmonic tweezer experiments. An inverse relation with a Pearson correlation coefficient of 0.82 is observed between experiments and computations, in support of the consistency of the two sets of data.

When trapped, PR65 undergoes a characteristic step as it enters the trap, with an increase in the noise amplitude, as shown in Fig. 5A. Once in the trap, the Brownian motion of the particle results in increased “noise” in the photodiode signal (transmission through the aperture, *T*, normalized to the pre-trap level). A histogram of this noise sampled after the trapping event is given in Fig. 5B. The stiffer the optical tweezer potential, the less motion that the particle undergoes (*42*), or the smaller the amplitude (or signal-RMSD) of the signal. The stiffness is proportional to the polarizability of the particle, which is higher for longer particles, so smaller deviation is expected when the protein is extended by a point mutation, and vice versa.

Figure 5E shows that the mutants S323L and F502W exhibit the smallest signal-RMSD among all mutants, which is indicative of an extension in the end-to-end distance of the trapped protein due to mutations, and this change in conformation is consistent with the preference observed in MD simulations for these mutants (see Fig. 4, respective panels E and G). Conversely, the mutant D315E distinguished in simulations by its smallest average end-to-end distance (Fig. 4D) exhibits the largest signal-RMSD in nanoaperture experiments. Figure 5F shows that the experimental (abscissa, normalized signal-RMSD) and computational (ordinate, average end-to-end distance) data generated for all mutants yield a correlation coefficient of 0.82.

Another quantity extracted from the detected transmission is the power spectral density presented in Fig. 5C. This has a corner frequency proportional to the trap stiffness divided by the hydrodynamic drag on the protein (*42*), and so it is expected to show the opposite trend of increasing with increasing length of the particle (opposite to the signal-RMSD). Figure 5D corroborates this behavior. These two quantities are listed in Table 2 (columns 5 and 6) for WT PR65 and all mutants.

**Fig. 6.**
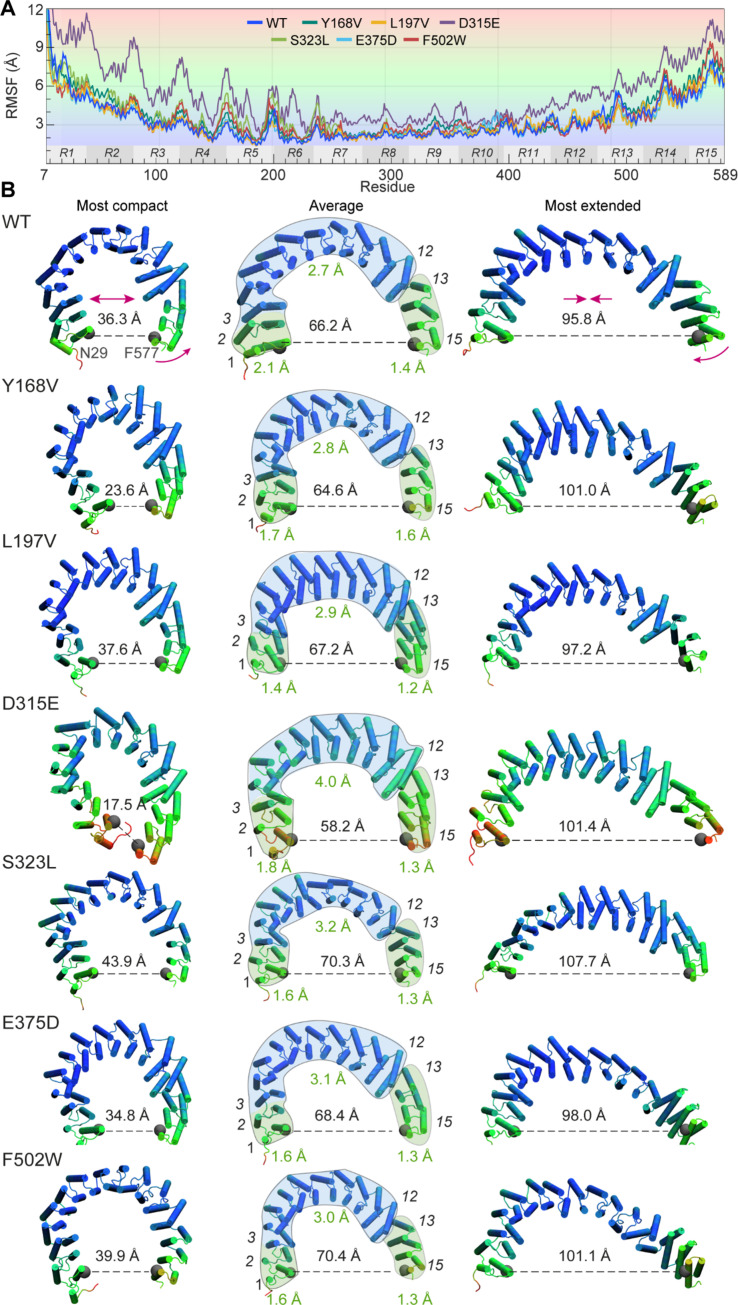
Residue mobilities observed for WT PR65 and its mutants. (**A**) RMSF profiles of PR65 residues observed in MD simulations for the WT PR65 and six mutants. (**B**) Conformations sampled during simulations. Each row illustrates the most compact (left), average (middle), and most extended (right) forms. The residues are colored in accordance with their RMSFs, as delineated in (A). The end-to-end distances are shown with a dashed line. The mean internal RMSD corresponding to each section (C-terminal, middle and N-terminal; separated by the green, blue, and green shades in the middle diagrams) is annotated in green. Internal RMSDs are obtained by separately superposing the three different sections. The C-terminal section shows relatively small internal RMSDs, while it undergoes large displacements with respect to the remaining portions, enabled by a rigid-like reorientation at the interface between repeats 12 and 13.

As noted above, S323L, E375D, and F502W shifted toward the extended conformation, and these showed the highest corner frequencies among all studied mutants as well as the WT PR65 (Fig. 5D). They also showed the lowest signal-RMSD (Fig. 5E); however, E375D was larger than the WT. We stress that this signal-RMSD is the result of Brownian motion of the protein in the optical potential, and therefore reflect both overall tumbling and global internal motions, different from the internal motions typically seen in MD simulations that occur at a much faster time scale, although the end-to-end fluctuations observed in MD and the extracted PCs reflect relatively slow events. Both of these findings separately confirm the in silico prediction from MD simulations that the protein is extended in the presence of the three mutations S323L, E375D, and F502W. The mutants L197V, Y168V, and D315E, on the other hand, showed the opposite trend in the experiments, and this confirms the MD predictions of a more compacted form (Fig. 5F). Finally, we note that the most dramatic behavior departing from the WT (and other mutants) has been observed in the mutant D315E, which is also consistent with MD results where D315E is distinguished by the impact of the point mutations on its structure and dynamics. Past works have investigated heating in similar aperture systems and found 1^°^C/mW (assuming a diffraction limited spot size of around 1 μm^2^) of laser power for the same geometry as our system (*54*–*56*). In our experiments, 16 mW of laser power was incident on the sample, and so the heating is expected to be around 16^°^C above room temperature, which is close to physiological conditions. We used the same laser power and wavelength for all trapping experiments to ensure consistency of the results.

### The interface between repeats 12 and 13 substantially contributes to the opening and closing of PR65

Figure 6 displays the root mean square fluctuation (RMSF) profile of residues (averaged over triplicate trajectories). The regular patterns of the repeat units can be distinguished. Figure 6B displays the mutants colored by their RMSFs, in line with the shades in Fig. 6A. Examination of the RMSFs shows that the structure can be divided into three substructures: a middle section composed of repeats 3 to 12, which shows small displacements (in blue), flanked by two segments (N-terminal repeats 1 and 2 and C-terminal repeats 13 to 15) that move substantially in space (in green).

Further examination of these individual sections shows that their spatial displacements do not necessarily reflect their conformational flexibilities. For example, although the middle section is subject to minimal motions, it undergoes substantial internal rearrangements or deformations, as measured by the RMSDs (between 2.7 and 4.0 Å) evaluated for each mutant {by structural alignment against compact PR65 [Protein Data Bank (PDB): 6NTS]}. These rearrangements are presumably required to accommodate the local conformational fluctuations with minimal effects on the flanking regions. In contrast, the N and C termini that substantially move in space show much smaller internal RMSDs indicative of en bloc movements of the repeats. In particular, the C-terminal section undergoes a rigid-like reorientation with respect to the middle section, enabled by hinge bending at the interface between repeats 12 and 13. The internal RMSDs within that section are confined to 1.2 to 1.6 Å. These rigid-body movements of the C-terminal section, combined with the local rearrangements of the remaining structure, enable the opening/closing of PR65 that may sample compact and extended forms, as illustrated in Fig. 6. Notably, F502W, at the inner helix (helix 2) of repeat 13 near this hinge region, considerably alters PR65 equilibrium structure in favor of more extended conformations, underscoring the mechanical significance of this particular site.

### The mutant F502W exhibits an increased ability to transition between open and closed forms, whereas D315E exhibits a decreased ability, compared to WT PR65

To further investigate the effect of these point mutations on PR65 structural dynamics, we evaluated the correlation cosines between the global movements observed during simulations and the change in structure experimentally observed between the compact and extended PR65. The global movements sampled in MD simulations were characterized by principal components analysis (PCA) (*57*). The PCA was performed using the triplicate MD trajectories (total of 1.962 μs) generated for WT PR65 and each of the six mutants. First, we aligned the MD conformations against the compact PR65 structure to exclude external (translational and rotational) motions. Thus, PCA yields 3*N*-6 internal motions. The first principal component (PC1) describes the most dominant mode of collective motion, which is also energetically the most favorable (soft) mode, succeeded by PC2 and PC3.

**Fig. 7.**
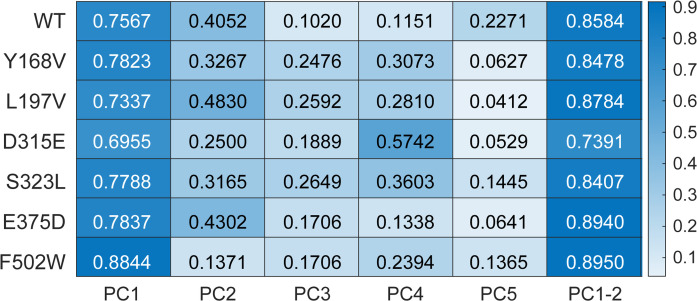
Correlation cosines between the PCs sampled in simulations and the deformation vector experimentally observed between the compact and extended forms of PR65. The correlation cosines between the top 5 PCs of the WT and mutants derived from the combined (triplicate) trajectories, and the deformation vector that delineates the transition from compact to extended PR65 structures. The *x* axis refers to PC1 to PC5, individually, and the last column shows the cumulative effects of PC1 and PC2. The correlation cosines are color-coded for clarity: White signifies no correlation, whereas blue indicates strong correlation.

Our goal was to assess whether the mutations impaired (or enhanced) the ability of PR65 to undergo its functional transitions between extended and compact forms. To this aim, we calculated the 3*N*-dimensional deformation vector pointing from the compact (PDB: 6NTS) to the extended (PDB: 1B3U) structures of PR65 and examined if/how the soft PCs derived from MD simulations correlated with this vector. As a quantitative measure, we used the correlation cosine between the PCs (from simulations) and the deformation vector (from experiments) (*16*). The heatmap in Fig. 7 presents the results. First, we note that the WT PR65’s PC1 yields a correlation cosine of 0.756 with the experimentally observed structural change, indicating this mode’s ability to facilitate the transition between the compact and extended PR65 structures. This is in accord with the previously reported intrinsic ability of PR65 to accommodate, if not drive, these functional changes. Then, we examined if the mutants retained the same ability or were relatively impaired. In the cases of D315E and L197V mutants, PC1’s ability to enable the conformational transition between the compact and extended forms decreased to 69.55% and 73.37%, respectively, indicating a small loss in functionality. In contrast, Y168V, S323L, and E375D yielded values of 78.23%, 77.88%, and 78.37%, respectively, suggesting that the PR65 global dynamics is robust to those point mutations. Strikingly, F502W stood out from other mutants, by yielding a correlation cosine of 0.884, which is even higher than that of the WT and points to a gain in function. This result draws attention to the significance of replacing F502 by a tryptophan on the functional dynamics of PR65 dynamics, which may have important ramifications for redesign or alteration of PP2A catalytic activity.

We further evaluated the cumulative correlation cosines with subset of two modes (PC1 and PC2 in Fig. 7, last column), in accord with our previous study (*58*). The cumulative correlation cosines for F502W PC1 and PC2 reach 0.895, which is higher than that of WT (0.858). D315E results in lower cumulative correlation cosines (0.739 in PC1 and PC2) than the WT. Therefore, our findings suggest that the single point mutation F502W could promote the ability of PR65 to undergo transitions between the compact and extended structures to accommodate trimeric assembly or diverse regulatory subunit binding. Conversely, D315E nudges the system toward a state that less favorably accommodates such transformations. The impact of D315E on PR65 structure and dynamics is also established in the highest normalized signal-RMSD and lowest corner frequency observed in nanoplasmonic tweezer experiments.

## DISCUSSION

PP2A is a heterotrimeric serine/threonine protein phosphatase that plays an essential role in maintaining cellular homeostasis, and its dysregulation has been linked to multiple types of cancer, Alzheimer’s disease, and increased susceptibility to pathogen infections. Its scaffolding subunit PR65 is an archetypal example of a TR protein. TR proteins often act as structural scaffolds, and structural and biophysical analysis of these proteins from diverse biological contexts has revealed extraordinary structural elasticity (*21*, *22*, *24*, *59*–*65*). In a recent review, we sought to understand this striking feature of TR proteins, and we showed that, through their evolutionarily optimized conformational mechanics, they act to facilitate the assembly and functional interactions of the complexes in which they participate (*66*). Notably, TR proteins are highly stable, and at the same time, they can sustain large changes in their conformations to enable structural rearrangements that are often functional. Our anisotropic network model (ANM) analysis (*66*) showed how TR proteins’ intrinsic dynamics facilitate functional complexation in phosphatases (*67*), chaperones (*68*), nuclear transporters (*69*), and transcriptional regulators (*70*). A recent study further showed how carefully selected single amino acid substitutions can alter the overall superhelical geometry of a TR protein (*65*). In the case of PR65, previous work showed that its structural fluctuations lead to cooperative expansions and contractions of its horseshoe-like structure (*16*). These fluctuations are crucial for optimally binding the catalytic and regulatory subunits as well as facilitating the allosteric communication across the PP2A heterotrimer. Previous experimental studies have indicated that PR65 mutations at specific positions can disrupt the binding of certain regulatory subunits (*17*–*19*), which in turn could lead to reduced or impaired suppressor activity and to cancer development.

Given the significance of PR65 conformational dynamics, we focused here on understanding how sensitive PR65 motions were to specific amino acids, primarily those occupying hinge sites that control their global dynamics. We explored how specific substitutions at those sites, which would not compromise the overall stability and function, might alter the structure and dynamics of PR65. Toward this goal, we undertook a multifaceted approach, composed of (i) in silico selection of candidate mutations at hinge sites, which would affect the dynamics without abolishing the stability or function, (ii) experimental selection of mutants after verification that they are expressed and soluble, (iii) MD simulations of the effects of these mutations on structure and dynamics, and (iv) validation of predicted changes in structure and dynamics with the help of nanoaperture optical tweezer experiments. The latter step was important in two respects: verifying that the changes observed in simulations were consistent with experimental observations and demonstrating the utility of this new technology for characterizing single-molecule structure and conformational fluctuations.

To ensure that we selected for experimental investigation only those mutants that affect the dynamics without abolishing stability, we first tested their expression levels in *E. coli*. It is well known that cells cannot produce proteins if their structures are too disrupted by mutations; there are many human disorders, for example, cystic fibrosis and lysosomal storage diseases and many rare diseases, where loss of protein expression correlates with mutant destabilizing effects, and many therapies are based on the principle of “pharmacological chaperones” to restore stability and thereby protein expression and function (*71*). Thus, protein expression tests make a reliable and fast readout of mutant stability. The experimentally observed expression levels of the mutant proteins in *E. coli* were consistent with the in silico predictions of pathogenicity. Notably, eight of nine mutants predicted by Rhapsody to be nonpathogenic were confirmed experimentally to be expressed and soluble (most of them in substantial amount). Conversely, of the 12 predicted to be pathogenic, none was expressed/soluble, except for L372V in small amounts. As to ΔΔ*G* values, 9 of 12 mutations predicted by PROTSPOM to strongly destabilize the protein had no detectable expression. Conversely, five of the six expected to retain stability were expressed. However, two mutations predicted to be mildly destabilizing were not expressed. These mutations might induce local or partial unfolding, which may have variable consequences on cellular protein levels (*72*). PROTSPOM results are subject to a root mean square error (RMSE) of 0.92 kcal/mol between predicted and experimental ΔΔ*G* values, hence the occasional lapses in stability estimates.

Our MD simulations revealed that S323L, E375D, and particularly F502W stabilized relatively more extended conformations, whereas Y168V and specifically D315E additionally favored compact forms. In addition, the mutations F502W and D315E affected the global dynamics of PR65 in contrasting ways: F502W enhanced the ability of PR65 to undergo its functional change in structure between compact and extended forms, suggesting a gain of function, whereas D315E had the opposite effect. We anticipate that such structural and dynamic changes could affect PP2A function, given the role of PR65 as a scaffold and its required structural flexibility for trimeric assembly. We leveraged the power of optical tweezers to analyze the changes in conformational flexibility of both the WT PR65 and its mutants at the single-molecule level. Unlike conventional optical tweezers that use labels and/or tethers, we used nanoplasmonic optical tweezers that allow for studying the unmodified protein at the single-molecule level without the use of labels or tethers. Comparison of the optical tweezer data obtained for PR65 mutants with those observed in the WT PR65 revealed trends consistent with the MD simulations. The mutants S323L and F502W showed substantially high corner frequencies along with low signal-RMSDs (Fig. 5 and Table 2), indicating that these mutations resulted in higher trap stiffnesses or had more extended/stretched conformations compared to WT PR65, in accord with the behavior observed in MD simulations. D315E, L197V, and Y168V, on the other hand, exhibited lower corner frequencies and higher signal-RMSDs than the WT PR65, indicative of potentially less extended or more variable conformers compared with WT PR65. In each of these cases, the three independent MD runs yielded distinctive histograms for end-to-end separations, consistent with the ability of these mutants to sample a multitude of conformational states (Fig. 4). Finally, E375D exhibited structural and dynamic features close to WT PR65, in both computations and experiments. Overall, a correlation coefficient of 0.82 between experimental and computational data (Fig. 5F and fig. S5) was obtained, which shows promise for this combined approach to detect the subtle changes in structure and dynamics induced by point mutations.

The findings here provide a better understanding of the changes in PR65 structure and dynamics in response to amino acid substitutions and provide new insights for rational modification or redesign of PR65 function. Integrated analysis with nanoaperture optical tweezer experiments helped establish/verify the role of specific residues in the conformational mechanics of PR65. Future exploration of the impact of point mutations on modulating the conformational space of PR65 may pave the way for the development of novel therapeutic strategies for diseases associated with PP2A dysfunction. By unraveling the complexities of PR65 and its role within the broader PP2A family, the study helps us move closer to unlocking the potential for targeted therapies and improved treatments for diseases linked to PP2A dysregulation.

Apart from results for PR65, the present study also illustrates how our recently developed in silico saturation mutagenesis screen combined with a GNM-based hinge site detection can help identify specific mutations that may alter function while retaining the fold, and how the effect of mutations on structure and conformational fluctuations can be assessed by a combination of computational (MD simulations) and experimental (plasmonic nanoaperture trapping) studies. It offers an integrated protocol for exploring the structural and dynamic consequences of mutations, generalizable to other systems. Building upon the established optical trapping approach, mmWave-THz dielectric spectroscopy emerges as a viable method for the real-time tracking of protein dipole movement and globular vibration modes. This technique offers the capability to investigate ultrafast dynamics implicated in extensive biomolecular conformational shifts on a broad scale while maintaining a noncontact and nonintrusive nature. Thus, it presents a compelling pathway for delving into the intricacies of protein conformational alterations.

## MATERIALS AND METHODS

### System and MD simulations

The PR65 structure resolved for the trimeric PP2A, deposited in the PDB (6NTS) (*3*), was used as input in our simulations. PR65 is in its compact form in the trimer. The structure was simulated in TIP3P explicit water, with 144 Å of water padding in all directions. Ions were added to neutralize the systems, and ion concentrations were set to 150 mM NACl. System size was ~286,830 atoms for WT PR65 simulations. All system preparations were performed in VMD, and all MD simulations were performed in NAMD3 (*73*) using the CHARMM36 all-atom additive protein force field (*74*). A time step of 2 fs was used in the simulations. Temperature was kept constant at 310 K via Langevin dynamics using a damping coefficient of 1 ps^−1^. The pressure was kept at 1 atm using the Langevin Nosé-Hoover method with an oscillation period of 100 fs and a damping time scale of 50 fs. A cutoff distance of 12 Å was adopted for van der Waals interactions. To calculate long-range electrostatic interactions, the particle-mesh Ewald method was used. PR65 mutants were generated by introducing single point mutations using the mutator plugin of VMD. PR65 mutants were simulated following the steps indicated above. Two rounds of minimization and equilibration simulations were performed before each production run. First, the protein was maintained in a fixed structure and the system was subjected to 10,000 energy minimization steps, followed by 1 ns of stabilization to equilibrate the solvent around the protein. Subsequently, a second round of minimization-equilibration was performed, where each system underwent an additional 10,000-step minimization, this time without any restrictions, which was subsequently followed by 4-ns stabilization using harmonic constraints (of 1 kcal mol^−1^ Å^−2^) on C^α^-atoms. Following these simulations, constraints were completely removed, and the system underwent 4 ns of equilibration, followed by production runs.

Conformations were recorded every 0.1 ns during MD simulations and used in RMSD and RMSF evaluations and in PCA. Thus, the three sets of runs of 19,620 snapshots based on a total of 3 × 654 ns = 1962 ns simulations were carried out for the WT PR65 and for each mutant (with a cumulative run time of 13.734 μs). We performed all calculations using our custom analysis codes, executed in VMD and MATLAB, which also used some of their built-in functions.

### In silico saturation mutagenesis

We used the Rhapsody (*32*, *35*) tool to carry out in silico saturation mutagenesis on the apo form of PR65 (PDB: 1B3U). Rhapsody was used to predict the pathogenicity corresponding to all possible single point mutations at each residue position of PR65. We further used PROTSPOM (*33*) to predict the change in Gibbs free energy of folding associated with all possible point mutations for each residue position.

### Hinge site detection

Hinge sites within a specific elastic network model (ENM) mode refer to regions that exhibit minimal displacements, if any, during that particular mode. Residues participating in these regions act as pivotal or anchor points, connecting substructures that move collectively around them, and as such, they play a crucial mechanical role. In the GNM analysis, the hinge sites are identified as the zero-crossover points in the mode shapes generated for each mode (*38*, *39*). The *i*th mode shape is obtained by plotting the elements of the *i*th eigenvector of the *N* × *N* connectivity/Kirchhoff matrix as a function of residue index for a protein of *N* residues (*38*, *39*). In our study, we specifically concentrated on the global hinges found in the soft (lowest frequency) GNM modes, e.g., modes 1 to 6 at the lower frequency range of the mode spectrum. We used the calcHinges function of ProDy (*75*) with the default parameters and protocol to compute the hinge sites corresponding to these global modes.

### Optical trapping experiments

To perform the optical trapping experiment, we first make a microwell on a clean glass microscope slide of 150 μm thickness (Ted Pella Inc.) using an imaging spacer (Secure Seal imaging spacer, Grace Bio-labs) to form an open chamber measuring 120 μm in depth and 9 mm in diameter. Using a micropipette, this chamber was filled with 10 μl of the analyte, diluted to 20 times its original concentration, and a sample of gold-on-glass, on which DNH apertures have been fabricated by colloidal lithography (*76*), was inverted over it, sealing off the chamber. The sample so prepared was placed on a sample stage between a 100× oil immersion objective and a 10× collection objective, with the side of the microscope cover slide in contact with the oil immersion objective. By turning the z-knob on a three-stage piezo-controller used to move the sample mounted on the sample stage, a focal position was reached whereupon multiple bright spots, corresponding to apertures on the gold-on-glass sample, show up on a computer screen connected to a CCD (charge-coupled device) camera, which captures light going through the apertures. The laser was then turned on to a very low drive current (18 mA, for example), and the x- and y-knobs of the piezo were used to move the gold-on-glass sample in a horizontal plane to select an aperture by aligning a white spot on the screen to the center of the laser’s diffraction pattern on the same screen. A half-wave plate (Thorlabs, WPH05M-980) mounted in the laser path was used to check whether the selected aperture was a DNH by rotating the polarization and observing the change in signal level of the avalanche photodiode (APD) collected by the USB-4771A data acquisition module. For a DNH, the change in signal level should be between 30% and 50% because the DNH has a polarized transmission, whereas a single nanohole and a triangular triple shows negligible change in the signal level with polarization rotation (*28*). To be specific, the APD typically reads around 1.6 V at the minimum transmission polarization and between 2.1 and 2.4 V at the maximum transmission polarization for a DNH. If an aperture failed this test, a different one was selected by the same procedure and the test was repeated until a DNH was found. The laser power was then increased to 22.5 mW before the 100× objective (corresponding to a drive current of 79 mA by our calibration), and identify trapping, observed as a discrete jump and an increase in noise of at least 10% on the screen connected to the signal acquisition module (as in Fig. 4A above). The data, acquired at a 100-kHz sampling rate, were then exported to MATLAB for analysis.

### Protein expression and purification

Site-specific mutations were introduced to the thrombin cleavable glutathione *S*-transferase (GST)–PR65–H_6_ fusion protein pRSETa plasmid using QuikChange site-directed mutagenesis protocol [Agilent (UK) Ltd.]. GST-tagged PR65 proteins (WT and mutants) were expressed in *E. coli* as described previously (*77*). In brief, plasmid encoding was transformed into chemically competent C41 *E. coli* (Komander laboratory, MRC-LMB, Cambridge). Cultures were grown at 37°C in 2xYT medium containing ampicillin (50 μg/ml) until an OD_600_ of 0.6 to 0.8 was reached. Protein expression was induced with 250 μM isopropyl-β-d-thiogalactopyranoside (IPTG) (Generon) at 25°C overnight. Cells were harvested by centrifugation at 4000*g* for 10 min at 4°C before resuspending in lysis buffer [50 mM tris-HCl (pH 7.5), 500 mM NaCl, 2 mM dithiothreitol (DTT)] supplemented with EDTA-free protease inhibitor cocktail (Sigma-Aldrich) and deoxyribonuclease (DNase) I (Sigma-Aldrich). The cells were lysed by passing the suspension two to three times through an Emulsiflex-C5 (AVESTIN) at pressures of 10,000 to 15,000 psi. Soluble protein was separated from cell debris and other insoluble fractions by centrifugation at 35,000*g* for 35 min at 4°C. The soluble protein fraction was applied to glutathione resin [Amintra Affinity, EGTA (0.5 g/liter), 2 mM DTT], the GST tag was cleaved with thrombin, and PR65 was eluted using a gravity column. After washing the column, the protein was subsequently eluted using a 20× column-volume salt gradient from 0 to 1 M NaCl. MonoQ fractions containing the protein were concentrated before application to a HiLoad 26/600 Superdex 200 pg (GE Healthcare) equilibrated in phosphate-buffered saline (pH 7.4) and 2 mM DTT. For small-scale expression tests, cells were cultured as described but in 5-ml volumes and induced with IPTG (3 hours, 37°C). Cells were centrifuged (8000*g*, 5 min) and lysed with BugBuster Master Mix (Merck KGaA, Germany). The soluble protein fraction was applied to glutathione resin [Amintra Affinity, EGTA (0.5 g/liter), 2 mM DTT] and washed with buffer [50 mM tris-HCl (pH 7.5), 150 mM NaCl, 2 mM DTT], and the protein was eluted [10 mM glutathione, 50 mM tris-HCl (pH 8), 2 mM DTT]. Samples were analyzed by SDS-PAGE (polyacrylamide gel electrophoresis) comparing the lysed, flowthrough, and eluted fractions.

### Protein stability measurements

Protein stability was measured using a thermal shift assay on a Bio-Rad CFX Connect qPCR instrument, whereby the unfolding is detected by fluorescence of the hydrophobic dye Spyro Orange that binds to the unfolded state of the protein. The experiments were performed in clear-bottom, half-volume, 96-well plates using final well volume of 25 μl, PR65 concentration of 10 μM, and Sypro Orange concentration of 0.025 μM. The samples were incubated at 25°C for 2 min before increasing the temperature by 0.5°C every 30 s up to 70°C. At each temperature, the fluorescence intensity was measured using an excitation wavelength of 471 nm and an emission wavelength of 570 nm. Data were analyzed using the GraphPad Prism software.
