# Supplementary material for: Influence of point mutations on PR65 conformational adaptability: Insights from molecular simulations and nanoaperture optical tweezers
Source: Sci Adv. 2024 May 31;10(22):eadn2208. doi: 10.1126/sciadv.adn2208 (PMC11141623; doi:10.1126/sciadv.adn2208)
Supplement: Supplementary file 1 — Supplementary Methods Figs. S1 to S5 Tables S1 to S3 [file sciadv.adn2208_sm.pdf]

Science Advances
AAAS

Supplementary Materials for

Influence of point mutations on PR65 conformational adaptability: Insights from molecular simulations and nanoaperture optical tweezers

Anupam Banerjee *et al.*

Corresponding author: Ivet Bahar, bahar@laufercenter.org; Reuven Gordon, rgordon@uvic.ca; Laura S. Itzhaki, lsi10@cam.ac.uk; Mert Gur, gurmert@pitt.edu

Sci. Adv. **10**, eadn2208 (2024)
DOI: 10.1126/sciadv.adn2208

This PDF file includes:

Supplementary Methods
Figs. S1 to S5
Tables S1 to S3

Nanoaperture Optical Tweezer-based Measurements

Optical tweezers use the momentum of photons to trap small particles. Nanoaperture optical trapping makes this process more suitable for trapping nanometric particles like single molecules by using an aperture in a metal film to confine light to a subwavelength volume below the diffraction limit, thereby reducing the trapping volume. This is beneficial for trapping single proteins. The nanoaperture also enhances the trapping efficiency because a nanoparticle will have a large influence on the transmission through the aperture because of dielectric loading effectively making the aperture larger: this process where the particle has an influence on the trapping potential is referred to as self-induced back-action. Shaped apertures, like DNH structures, allow for sharper field confinement and thereby enable trapping of smaller objects, like single proteins.

The DNH optical tweezer system combines a laser microcroscope, which is based on the Thorlabs optical tweezer kit, with an avalanche photodetector. The avalanche photodetector monitors the changes in the optical transmission through the aperture which can be used to detect the particle, but also the motion of the particle in the aperture due to Brownian or conformational changes. Elongated particles typically have larger polarizability, and so this makes there scattering larger (larger RMSD detected), but also creates a stiffer trapping potential so their corner frequency increases. Conventional optical tweezers have a corner frequency in the fluctuations (e.g., from displacement) that scales as the trap stiffness divided by the hydrodynamic drag. The same dependence has been used to characterize particles in a DNH optical tweezer setup *(50, 52)*.
In this work, all the proteins studied have nominally the same molecular weight and so the changes in polarizability from elongation are found from changes in the RMSD and corner frequency.

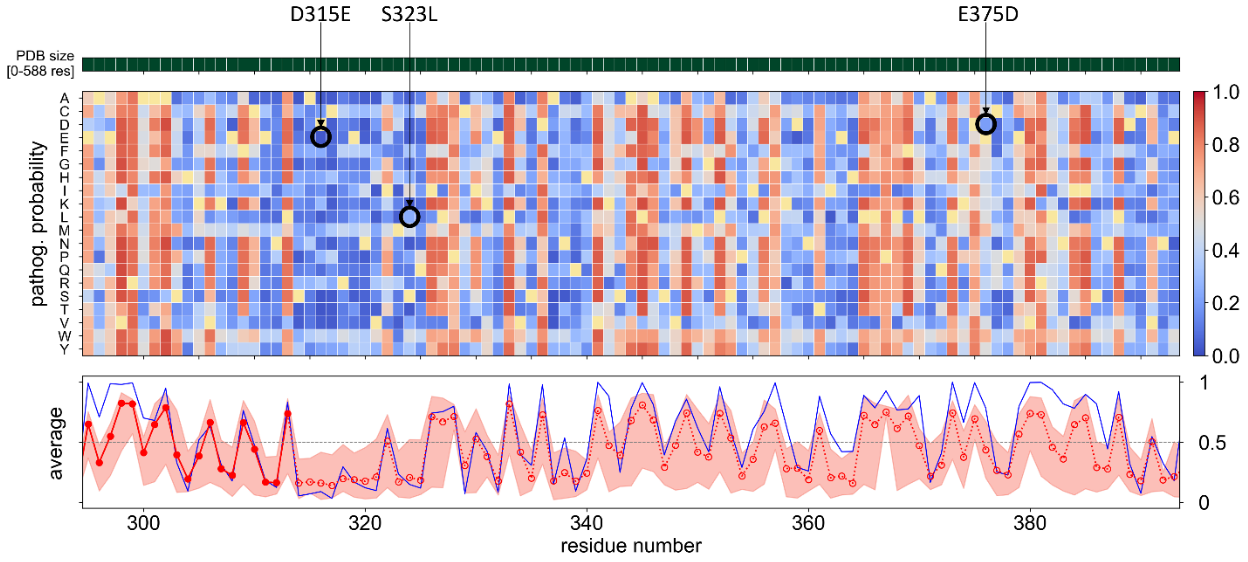

Figure S1. *In silico* saturation mutagenesis map generated for PR65 residues 296-394. Residues (along the abscissa) are color-coded according to their pathogenicity probability associated with all possible 19 mutations (ordinate), as predicted by Rhapsody *(32, 35)* for each residue. The wt residue is shown in cream. The color-coded entries represent the probability of having a pathogenic effect, with the scores 0 and 1 corresponding to fully neutral (*blue*) and fully pathogenic (*red*) effects. The mutations D315E, S323L and E375D are indicated by *black* circles. The curve underneath represents the averages over all entries in the corresponding column for each sequence position, providing a measure of the overall pathogenicity potential of each residue (red dots) regardless of the specific substitution. The pink shade provides the variance in the pathogenicity score (among all 19 substitutions) for each position, and the blue curve represents the result from PolyPhen-2 *(36)*. The entire pathogenicity profile for all PR65 residues can be obtained by running saturation mutagenesis on PR65 at the Rhapsody webserver (http://rhapsody.csb.pitt.edu/sat_mutagen.php).

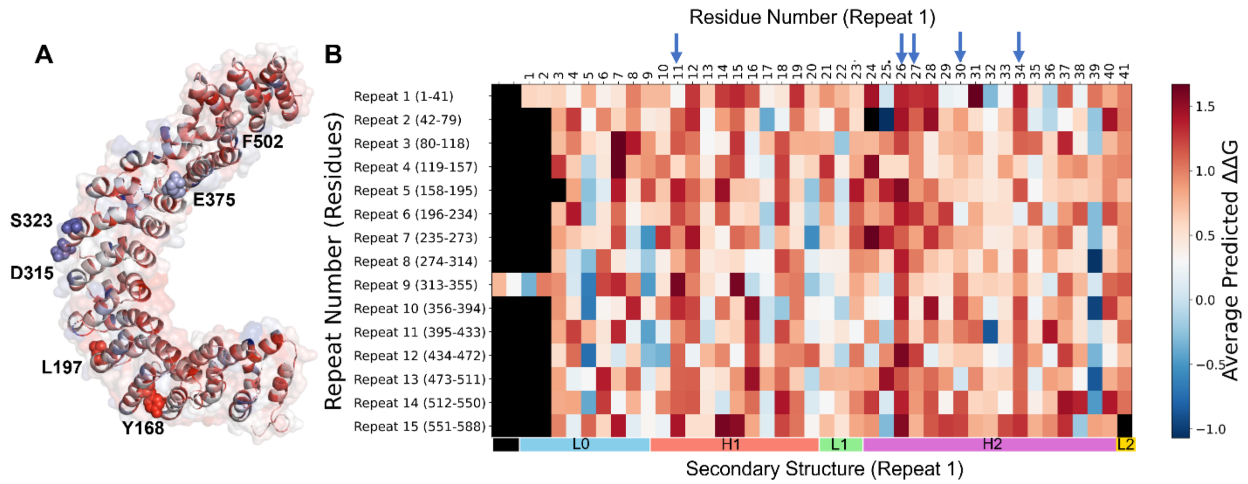

Figure S2. PROTSPOM-predicted average change ΔΔG folding free energy, across all single point mutations for each residue of PR65. (A) The apo form of PR65 color-coded by the average ΔΔG of all possible mutations as predicted by PROTSPOM *(33)* for each residue, and **(B)** corresponding heat map. The color varies from *deep blue* (maximal stabilization by -1.07 kcal/mol) to *red* (maximal destabilization by1.67 kcal/mol) induced by mutations (averaged over all 19 substitutions) for each position (*abscissa*) along each repeat unit (*ordinate*) of PR65.

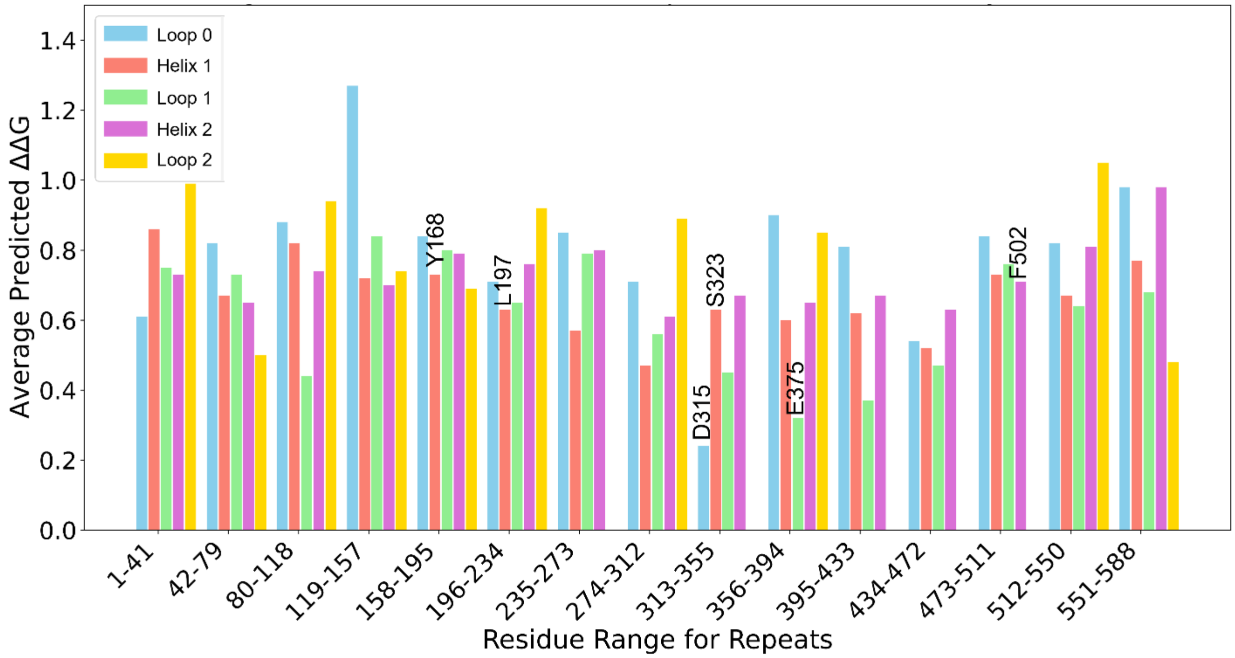

Figure S3. PROTSPOM-predicted ΔΔG values across secondary structures for the 15 HEAT repeats of PR65.

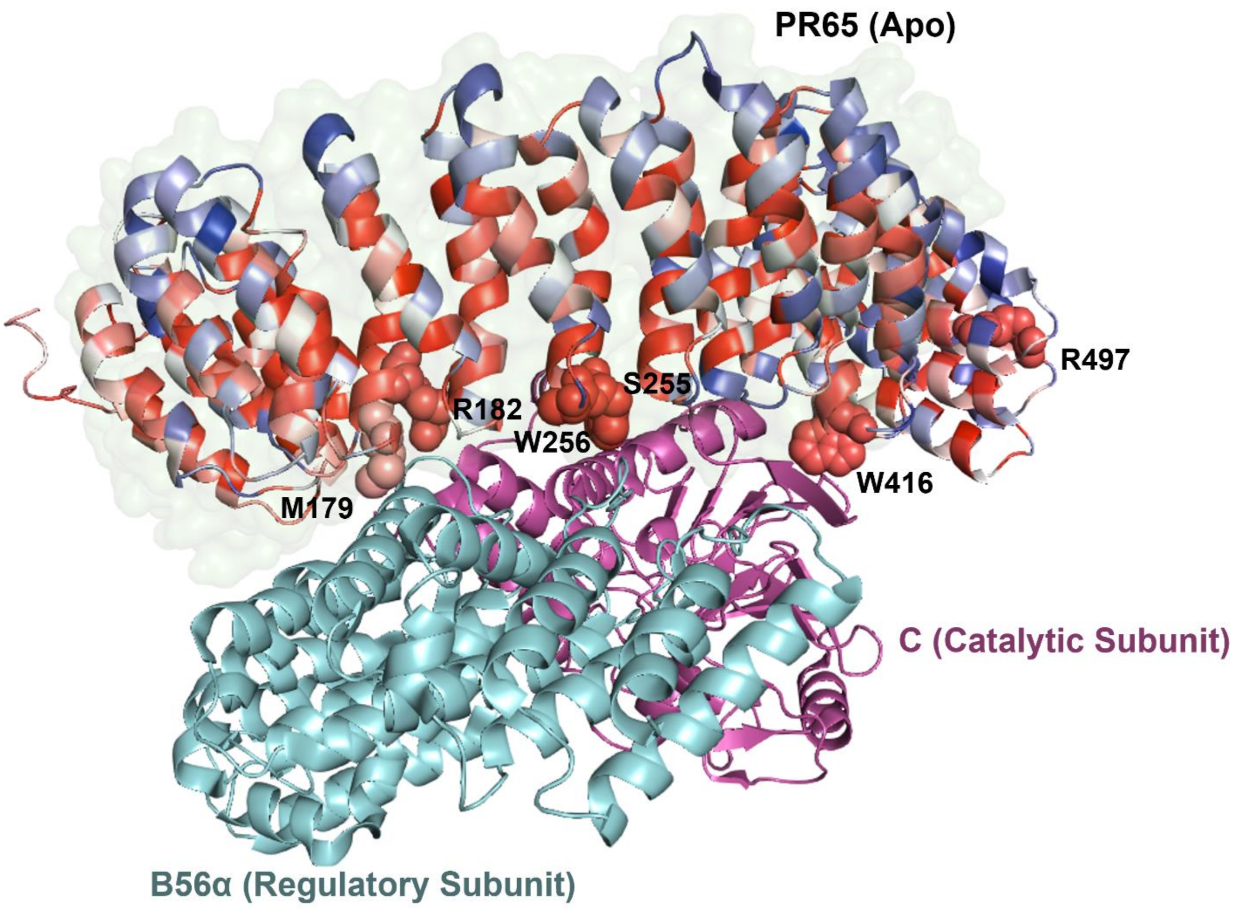

Figure S4. Maximally destabilizing and pathogenic point mutations of PR65 occur at the interface between the catalytic and regulatory subunits. The PR65 mutations M179H (ΔΔG = 1.52 kcal/mol, pathogenicity probability = 0.71), R182T (ΔΔG = 1.46 kcal/mol, pathogenicity = 0.77), S255F (ΔΔG = 1.47 kcal/mol, pathogenicity = 0.86), W256H (ΔΔG = 2.09, pathogenicity = 0.82) at the interface with the regulatory subunit, and the mutations W416F (ΔΔG = 1.23 kcal/mol, pathogenicity = 0.82), R497T (ΔΔG = 1.34 kcal/mol, pathogenicity = 0.81) at the interface with the catalytic subunit are distinguished in Rhapsody and PROTSPOM analyses to be strongly destabilizing and potentially pathogenic.

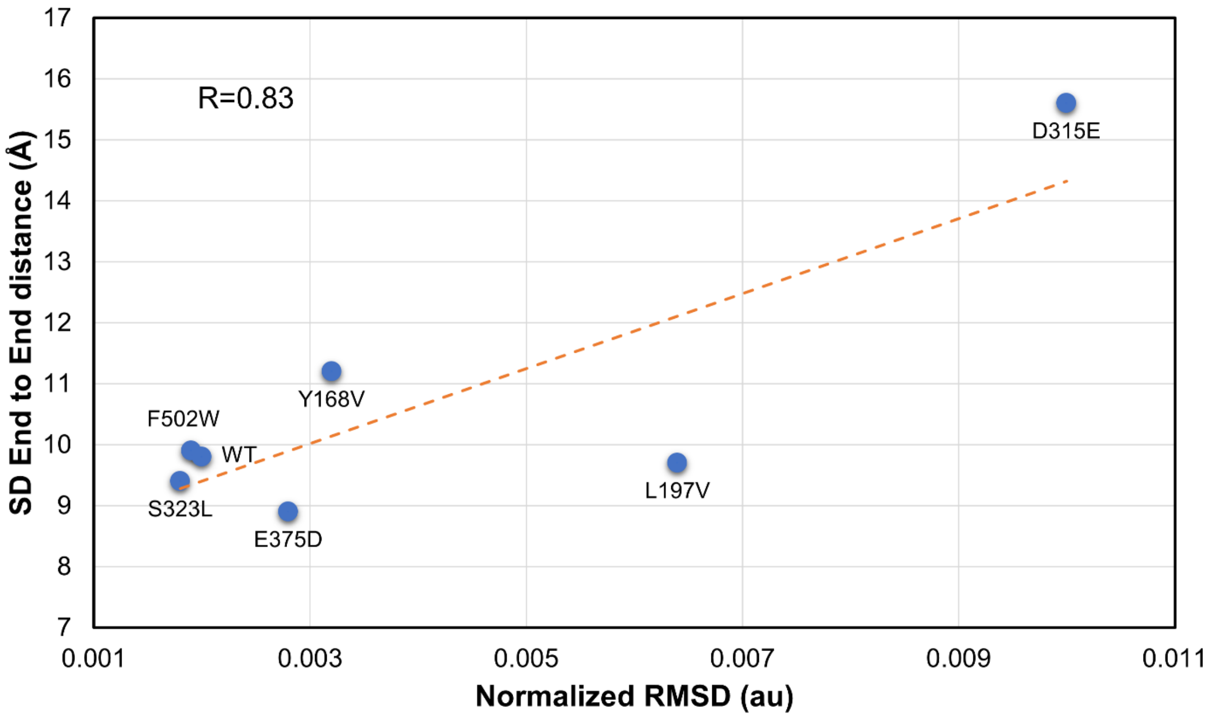

Figure S5. Standard deviation in PR65 end-to-end distance is correlated with the experimentally measured normalized signal-RMSD for the six mutants.

Table S1. Residue ranges in different structural elements of the 15 HEAT repeats in PR65 (PDB: 1B3U)

**HEAT Repeat**	**Loop 0**	**Helix 1**	**Loop 1**	**Helix 2**	**Loop 2**
1 (1-41)	1-9	10-20	21-23	24-40	41-41
2 (42-79)	42-42	43-57	58-61	62-73	74-79
3 (80-118)	80-84	85-97	98-100	101-117	118-118
4 (119-157)	119-119	120-136	137-139	140-150	151-157
5 (158-195)	158-158	159-174	175-177	178-194	195-195
6 (196-234)	196-196	197-213	214-216	217-233	234-234
7 (235-273)	235-235	236-252	253-255	256-273	-
8 (274-312)	274-274	275-291	292-294	295-311	312-312
9 (313-355)	313-315	316-334	335-337	338-355	-
10 (356-394)	356-356	357-373	374-376	377-393	394-394
11 (395-433)	395-395	396-412	413-415	416-433	-
12 (434-472)	434-434	435-451	452-454	455-472	-
13 (473-511)	473-473	474-490	491-493	492-511	-
14 (512-550)	512-512	513-529	530-532	533-549	550-550
15 (551-588)	551-551	552-568	569-571	572-585	586-588

Table S2. Computational and experimental characterization of selected mutations at PR65 hinge sites(*)

Repeat	Mutant	Pathogenicity prob [0-1][1]	ΔΔG (kcal/mol) [-1.49-2.21][2]	Hinge Mode	Solvent accessibility [0-9][3]	Seq conservation [1-9][4]	Mutation group	Experimentally observed expression or solubility
2	L72S (Helix 2)	0.89	1.62	[4]	0	8	a	No soluble protein
3	L111S (Helix 2)	0.86	1.49	[3]	0	6	a	No soluble protein
3	I114N (Helix 2)	0.82	1.56	[3]	0	7	a	No soluble protein
**5**	**Y168V (Helix 1)**	**0.18**	**1.95**	**[2]**	**2**	**1**	**b3**	**Significant amount of soluble protein**
5	R182T (Helix 2)	0.77	1.46	[5]	3	8	a	No soluble protein
5	E190W (Helix 2)	0.61	0.68	[5]	2	1	a	No expression (no band on SDS PAGE in soluble or insoluble fractions)
**6**	**L197V (Helix 1)**	**0.04**	**1.78**	**[6]**	**4**	**1**	**b3**	**Significant amount of soluble protein**
7	Y260D (Helix 2)	0.61	1.59	[6]	3	6	a	No soluble protein (insoluble band only)
8	R298T (Helix 2)	0.77	1.53	[3]	1	9	a	No soluble protein
**8**	**E310L (Helix 2)**	**0.10**	**-1.47**	**[1]**	**3**	**1**	**b1**	**Small amount of soluble protein**
**9**	**D315E (Loop 0)**	**0.03**	**-0.67**	**[1]**	**6**	**1**	**b1**	**Significant amount of soluble protein**
**9**	**S323L (Helix 1)**	**0.24**	**-1.16**	**[1]**	**5**	**1**	**b1**	**Significant amount of soluble protein**
10	L372V (Helix 1)	0.70	1.65	[4]	0	9	a	Small amount of soluble protein
**10**	**E375D (Loop 1)**	**0.20**	**-0.60**	**[4]**	**7**	**5**	**b1**	**Significant amount of soluble protein**
10	L381W (Helix 2)	0.63	1.62	[4]	3	7	a	No expression
10	I383G (Helix 2)	0.83	1.54	[4]	0	8	a	No expression
**10**	**E392Q (Helix 2)**	**0.14**	**-1.34**	**[4]**	**7**	**1**	**b1**	**No expression**
**12**	**E440Q (Helix 1)**	**0.14**	**-0.61**	**[2]**	**5**	**4**	**b1**	**Small amount of soluble protein**

12	L445G (Helix 1)	0.75	1.49	[2]	0	6	a	No expression
**13**	**F502W (Helix 2)**	**0.36**	**1.09**	**[3]**	**2**	**4**	**b2**	**Significant amount of soluble protein**
14	K541S (Helix 2)	0.64	1.09	[6]	3	7	a	No soluble protein

[1] *ΔΔG values computed using PROTSPOM (33), represent the effect of mutation on folding stability, more positive values suggesting more destabilizing mutations. ΔΔG < 0 kcal/mol is considered stabilizing, 0 ≤ ΔΔG ≤ 1.25 kcal/mol is mildly destabilizing and ΔΔG > 1.25 kcal/mol is considered to be destabilizing*

[2] *Pathogenicity probability computed using Rhapsody (32, 35). The probabilities vary in the range [0-1], with higher values indicating higher probability of having a pathogenic effect.*

[3] *Solvent accessibility computed using STRIDE (40). A higher value suggests higher accessibility to water.*

[4] *Residue conservation score computed using ConSurf (41). Values vary in the range 1 (least conserved or highly variable) to 9 (fully conserved).*

(*) Rows highlighted in bold indicate the mutations identified by Rhapsody to be non-pathogenic. Others (score > 0.60) were predicted to be pathogenic. Note that in all such cases experiments indicated that there was no soluble protein or no expression of protein, except for one case (L372V) where there was a small amount of soluble protein.

Table S3. Experimentally determined melting temperatures of PR65 WT and mutants. Mean and standard deviation are listed from technical triplicates.

**Mutation**	**Melting temperature (°C)**
WT	51.3± 0.1
Y168V	50.9± 0.2
L197V	50.4± 0.3
D315E	50.4± 0.3
S323L	49.7± 0.2
E375D	51.0± 0.2
F502W	51.9± 0.2
